# Serum Procalcitonin in Culture-Confirmed Melioidosis: A Systematic Review and Meta-Analysis with Narrative Evaluation of Clinical and Prognostic Implications

**DOI:** 10.3390/diseases14040119

**Published:** 2026-03-26

**Authors:** Jongkonnee Thanasai, Chaimongkhon Chanthot, Anchalee Chittamma, Supphachoke Khemla, Atthaphong Phongphithakchai, Moragot Chatatikun, Jitbanjong Tangpong, Sa-ngob Laklaeng, Wiyada Kwanhian Klangbud

**Affiliations:** 1 Faculty of Medicine, Mahasarakham University, Mahasarakham 44000, Thailand; jongkonnee@msu.ac.th; 2 Project for the Establishment of the Faculty of Medicine, Nakhon Phanom University, Nakhon Phanom 48000, Thailand; chaimongkhon251269@gmail.com; 3 Department of Pathology, Faculty of Medicine Ramathibodi Hospital, Mahidol University, Bangkok 10400, Thailand; anchalee.chi@mahidol.ac.th; 4 Division of Infectious Diseases, Department of Internal Medicine, Nakhon Phanom Hospital, Nakhon Phanom 48000, Thailand; 5 Nephrology Unit, Division of Internal Medicine, Faculty of Medicine, Prince of Songkla University, Songkhla 90110, Thailand; 6 School of Allied Health Sciences, Walailak University, Nakhon Si Thammarat 80160, Thailandsumoun2528@gmail.com (S.-n.L.); 7 Research Excellence Center for Innovation and Health Products (RECIHP), Walailak University, Nakhon Si Thammarat 80160, Thailand; 8 Medical Technology Program, Faculty of Science, Nakhon Phanom University, Nakhon Phanom 48000, Thailand; 9 Faculty of Medicine, Nakhon Phanom University, Nakhon Phanom 48000, Thailand

**Keywords:** melioidosis, *Burkholderia pseudomallei*, procalcitonin, biomarker, sepsis

## Abstract

**Background:** Procalcitonin (PCT) is a biomarker of bacterial infection and sepsis severity, but its role in melioidosis remains unclear. This study aimed to synthesize available evidence on serum PCT levels in culture-confirmed melioidosis and explore associations with disease severity and mortality. **Methods:** We conducted a systematic review following PRISMA guidelines and registered the protocol with PROSPERO (CRD420251166979). PubMed, Embase, and Scopus were searched up to 30 October 2025. Observational studies reporting serum PCT levels in microbiologically confirmed melioidosis were included. Study quality was assessed using the Newcastle–Ottawa Scale (NOS) for observational studies. Random-effects models were used to calculate pooled mean PCT levels, with heterogeneity assessed by *I*^2^. Sensitivity analyses were performed to explore the influence of historical and small-sample studies. **Results:** Seven studies comprising 284 patients with culture-confirmed melioidosis were included. The pooled mean PCT level was 14.46 ng/mL (95% CI: 4.59–24.33), with substantial heterogeneity (*I*^2^ = 87.7%). Sensitivity analyses excluding the oldest study and the smallest sample size reduced heterogeneity but retained consistently elevated PCT levels across cohorts. Higher PCT concentrations were consistently observed among patients with septic shock, bacteremia, and fatal outcomes, although variability in definitions precluded quantitative synthesis of prognostic effect sizes. These findings were based on heterogeneous study-level comparisons and could not be synthesized quantitatively. **Conclusions:** PCT is markedly elevated in melioidosis and reflects the severity of systemic infection, supporting its potential role as an adjunctive biomarker for early risk stratification. However, substantial heterogeneity and limited sample sizes prevent the establishment of a melioidosis-specific prognostic threshold. Standardized, prospective, multicenter studies are required to clarify the independent prognostic value of PCT in melioidosis management. This study establishes a pooled estimate of serum PCT levels in melioidosis and demonstrates that these values are comparable to those observed in severe bacterial sepsis, supporting its interpretation as a marker of systemic inflammatory burden rather than a disease-specific biomarker.

## 1. Introduction

Melioidosis is a severe infectious disease caused by the environmental Gram-negative bacterium *Burkholderia pseudomallei*, which is endemic in Southeast Asia and northern Australia, and increasingly recognized in other tropical and subtropical regions. The infection is typically acquired through percutaneous inoculation, inhalation, or ingestion, especially among individuals with occupational exposure to wet soil and surface water [1]. Clinically, melioidosis presents a wide spectrum of manifestations, ranging from localized skin infections to pneumonia, deep organ abscesses, and fulminant septic shock, with mortality rates often exceeding 40% in low- and middle-income countries [2,3]. This high case fatality rate is attributed to diagnostic delays, limited access to intensive care, and the high prevalence of comorbidities such as diabetes mellitus, which predispose individuals to severe disease [4]. Consequently, early identification of patients at the greatest risk of deterioration remains a clinical priority to guide timely intervention and optimize the use of scarce critical care resources.

Among the various biomarkers investigated for sepsis management, procalcitonin (PCT) has gained prominence as a promising indicator of bacterial infection severity and treatment response. PCT, a precursor of calcitonin produced by extra-thyroidal tissues in response to systemic bacterial infection, is widely used in sepsis for diagnosis, severity assessment, and antibiotic stewardship [5]. However, its role in melioidosis is less clearly defined due to the complex immunopathogenesis of *B. pseudomallei*, a facultative intracellular organism capable of eliciting a mixed inflammatory and immune response. The earliest major study on PCT in melioidosis by Smith et al. (1995) demonstrated markedly elevated PCT levels among patients with severe disease, reporting median concentrations of 350 ng/mL in non-survivors compared with 19 ng/mL in survivors [6]. This suggested a strong correlation between PCT elevation and mortality. In a more recent cohort study from northern Hainan, China, Zheng et al. (2023) reported a median PCT level of 1.31 ng/mL, significantly higher in septic and bacteremic cases [1]. Yet, differences in assay types and disease definitions limited cross-study comparability. Similarly, Nisarg et al. (2024) observed elevated PCT and CRP values among melioidosis non-survivors but identified low serum albumin and vasopressor requirement, rather than PCT, as independent predictors of 28-day mortality [3]. Additional studies have explored related biomarkers such as interleukin-6 (IL-6) and C-reactive protein (CRP), both of which showed prognostic associations with sepsis severity but limited specificity for melioidosis [5,7].

Despite these advances, key knowledge gaps persist. First, there is no standardized reference range for PCT in culture-confirmed melioidosis, which complicates clinical interpretation across settings. Second, the prognostic utility of baseline and serial PCT measurements remains unclear relative to other inflammatory biomarkers such as CRP and IL-6. Third, there has been limited integration of PCT into multivariable prognostic models, with most studies relying on small, single-center datasets and lacking adjustment for confounders [8]. These limitations hinder the ability to use PCT as a reliable risk stratification tool, particularly in resource-limited endemic regions where melioidosis outcomes are most severe.

To date, no study has quantitatively synthesized serum procalcitonin levels in culture-confirmed melioidosis. Establishing the expected magnitude of PCT elevation is important for clinical interpretation, particularly in endemic settings where severe sepsis is common. In addition, it remains unclear whether PCT behaves differently in melioidosis compared with other forms of Gram-negative sepsis or whether it provides disease-specific diagnostic or prognostic value.

To address these gaps, the present study undertakes a systematic review and meta-analysis to synthesize the available evidence on PCT levels in culture-confirmed melioidosis. The primary objective is to quantify typical serum PCT concentrations and provide a pooled benchmark to aid interpretation in endemic settings. The secondary objective is to examine the association between baseline PCT levels and adverse clinical outcomes, particularly mortality and severe disease, while critically appraising whether PCT has been effectively integrated into prognostic models. By consolidating evidence from diverse observational studies, this review aims to clarify the prognostic significance of PCT in melioidosis and establish its potential value as a biomarker for early risk stratification in this neglected but life-threatening tropical infection.

## 2. Materials and Methods

### 2.1. Protocol and Registration

This systematic review was conducted according to PRISMA 2020 guidelines [9] and registered in PROSPERO (registration number: CRD420251166979). Although the original protocol planned inclusion of randomized studies without formal quantitative synthesis, no randomized trials evaluating procalcitonin (PCT) in melioidosis were identified. Therefore, observational studies reporting extractable quantitative PCT data were included, and a random-effects meta-analysis was conducted. These protocol deviations were implemented to maximize available evidence and are reported transparently.

### 2.2. Search Strategy

A comprehensive literature search was performed on 30 October 2025 using three electronic databases: PubMed, Embase, and Scopus. The search strategy combined keywords and MeSH terms related to melioidosis and procalcitonin, including “melioidosis,” “*Burkholderia pseudomallei*,” “Whitmore disease,” “procalcitonin,” and “PCT.” The full search strategies for each database are provided in Supplementary File S1. No restrictions were applied to the study design, publication year, or geographic region.

### 2.3. Eligibility Criteria and Selection

Studies were eligible for inclusion if they enrolled patients with culture-confirmed melioidosis and reported quantitative serum procalcitonin (PCT) values. Both observational and interventional study designs were considered, provided that extractable numerical data were available. To ensure comparability across studies, only investigations reporting baseline or admission PCT measurements were included in the quantitative synthesis. Studies were excluded if they were reviews, narrative reports, or case reports and small case series including fewer than five patients. In addition, studies without extractable numerical PCT data and those conducted exclusively in animal models or laboratory settings were excluded. Two reviewers independently screened titles/abstracts and full texts. Disagreements were resolved by consensus.

### 2.4. Data Extraction

Data extraction was performed using a standardized form to ensure consistency across studies. The following information was collected from each eligible study: study characteristics (including author, year of publication, country, and study design), sample size, details of the procalcitonin (PCT) assay used, and the timing of biomarker measurement, with preference given to baseline or admission values. Quantitative PCT data were extracted as reported, including mean ± standard deviation (SD) or median with interquartile range (IQR), as applicable. Where available, data on mortality and disease severity outcomes were also extracted to allow narrative assessment of prognostic associations. The Wan et al. [10] method was applied to studies reporting PCT values as medians with interquartile ranges, specifically Zheng et al. (2023) [1], Nisarg et al. (2024) [3], and Smith et al. (1995) [6]. Extracted data used for the meta-analysis are provided in Supplementary Table S1.

### 2.5. Study Quality

The methodological quality of included studies was assessed using the Newcastle–Ottawa Scale (NOS) for observational studies. This tool evaluates studies across three domains, selection of participants, comparability of study groups, and outcome assessment, with a maximum score of nine stars. For cohort studies, we assessed representativeness of the sample, confirmation of melioidosis diagnosis, control of key confounders (e.g., age or comorbidities), and adequacy of outcome measurement and follow-up. Two reviewers independently performed the assessment, and disagreements were resolved by consensus.

### 2.6. Statistical Analysis

Pooled mean serum PCT levels were calculated using a random-effects model. Meta-analysis of raw means (MRAW) refers to the pooling of untransformed mean values and standard deviations across studies using inverse-variance weighting under a random-effects model. Statistical heterogeneity was quantified using the *I*^2^ statistic. Sensitivity analyses were performed to examine the influence of specific studies (e.g., historical cohorts or small sample sizes) on pooled effect estimates. Potential publication bias was assessed through visual inspection of funnel plots and Egger’s regression test.

Although procalcitonin values are typically right-skewed and may follow a log-normal distribution, consistent log-transformation across studies was not feasible due to heterogeneous reporting formats, including mixed use of means, medians, and incomplete dispersion data. Therefore, pooled raw means were calculated to maximize inclusion of available evidence, and findings were interpreted cautiously. In this context, geometric means and log-transformed analyses generally provide a more appropriate representation of central tendency and dispersion for skewed biomarker data. Such approaches were considered; however, consistent application across studies was not feasible without introducing additional assumptions.

Given the limited number of available studies in this rare disease context, small and specialized cohorts were included to avoid loss of relevant data. The influence of such studies was evaluated through sensitivity analyses.

All statistical analyses were performed using R software (version 4.4.2; R Foundation for Statistical Computing, Vienna, Austria) with the ‘*meta*’ and ‘*metafor*’ packages.

## 3. Results

### 3.1. Search Results and Study Selection

**Figure 1:**
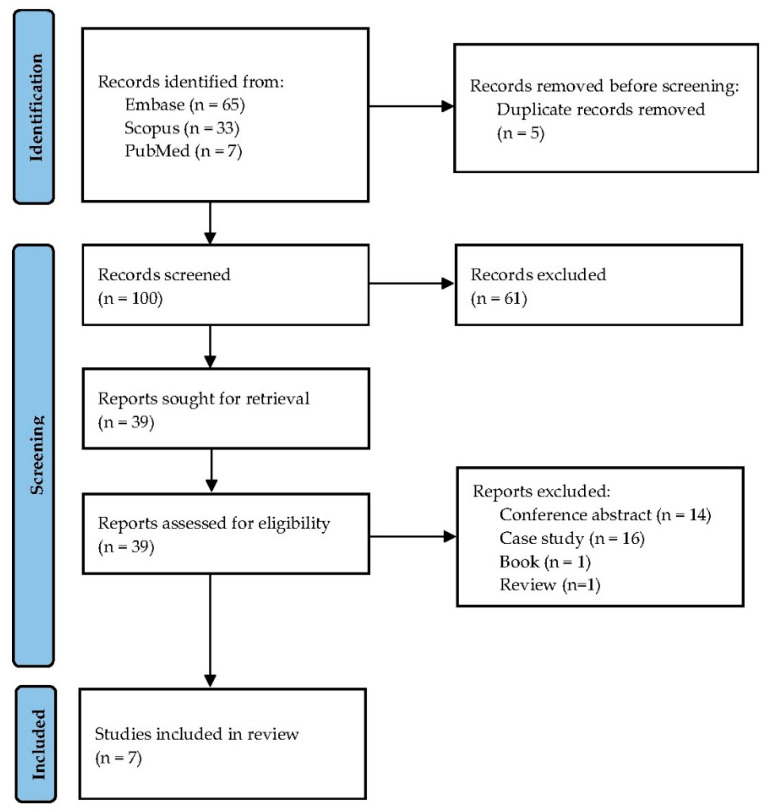
PRISMA Flow.

The literature search yielded 105 records: 65 from Embase, 33 from Scopus, and 7 from PubMed. After removing 5 duplicate entries, a total of 100 records were screened by title and abstract. Of these, 61 records were excluded for not meeting the eligibility criteria. Full-text assessment was conducted for 39 articles, of which 32 were excluded for the following reasons: conference abstracts (*n* = 14), case reports (*n* = 16), book (*n* = 1), and review article (*n* = 1). Ultimately, 7 studies met the inclusion criteria and were incorporated into the systematic review and meta-analysis. The study selection process is illustrated in Figure 1.

### 3.2. Characteristics of the Included Studies

A total of seven studies, published between 1995 and 2025, were included in this review. Most studies were conducted in melioidosis-endemic regions across Asia: three from India, two from Thailand, one from China, and one multi-center study involving participants from Cambodia, the Democratic Republic of the Congo, Nepal, and Sudan. All included studies employed observational designs, comprising four retrospective cohort or observational studies and three prospective observational studies. Sample sizes for patients with confirmed melioidosis ranged from 5 to 90 individuals, with the majority of studies enrolling more than ten participants.

Procalcitonin (PCT) was measured using a variety of assay platforms across the included studies. The earliest cohort from Thailand (Smith et al., 1995) employed an immunoluminometric assay [6], while the Thai biomarker study by Kaewarpai et al. (2023) utilized ELISA [11]. Several prospective studies assessing PCT alongside other sepsis-related biomarkers used TRACE (Time-Resolved Amplified Cryptate Emission) technology. In contrast, the specific assay type was not reported in several of the more recent retrospective cohorts.

Across the included studies, PCT served multiple clinical roles. In some cohorts, it was used primarily as an inflammatory marker within specific clinical presentations, such as osteoarticular melioidosis. In others, PCT formed part of a broader sepsis biomarker panel or was evaluated explicitly as a candidate prognostic marker. Elevated PCT levels were consistently associated with more severe disease [6]. Other studies demonstrated higher PCT concentrations in *Burkholderia pseudomallei* infections compared with other *Burkholderia* species, and in patients presenting with septic shock or fatal outcomes [8]. In the largest prospective cohort, PCT was evaluated alongside IL-1R2 and additional biomarkers for the development of mortality prediction models, whereas other studies incorporated PCT into broader clinical feature analyses rather than as a standalone predictor. A summary of key study characteristics is presented in Table 1.

### 3.3. Prognostic Association of PCT

Across included studies, higher serum PCT concentrations were consistently associated with greater disease severity and mortality. For example, Smith et al. demonstrated markedly higher admission PCT levels among patients who died compared with survivors (median: 350 ng/mL vs. 19 ng/mL), and levels above 100 ng/mL were strongly associated with mortality risk [6]. Similarly, cohort studies reported progressive increases in PCT with septicemia and multiorgan involvement. However, heterogeneity in outcome definitions and reporting prevented a pooled prognostic meta-analysis. Overall evidence suggests that PCT reflects inflammatory burden rather than disease specificity.

### 3.4. Quality Assessment

The methodological quality of the included studies, as assessed using the Newcastle–Ottawa Scale (Supplementary Table S2), was generally moderate to high. Overall NOS scores ranged from 6 to 8 out of a maximum of 9 stars. Most studies achieved high scores in the selection and outcome domains, reflecting appropriate case definition (culture-confirmed melioidosis) and adequate outcome assessment. However, comparability scores were limited in several studies due to insufficient adjustment for potential confounders such as age, comorbidities, or disease severity. No study was judged to be at a high risk of bias overall, although the observational design of all included studies inherently limits causal inference.

### 3.5. Quantitative Synthesis (Meta-Analysis)

#### 3.5.1. Pooled Mean Procalcitonin Levels

**Figure 2:**
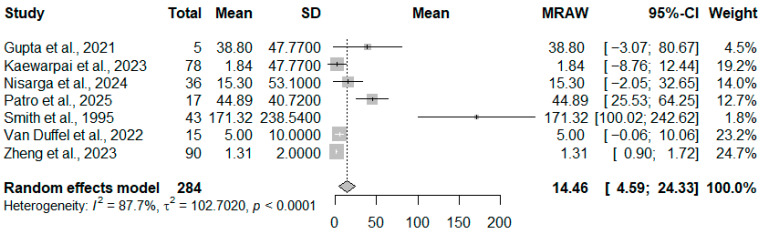
Forest plot of the seven included studies. The studies included are [1,3,6,8,11,12,13]. Squares represent individual study effect sizes, with horizontal lines indicating 95% confidence intervals. The size of each square reflects the study weight, and the diamond represents the pooled estimate from the random-effects model. Bold text is used to highlight the overall summary estimate and does not indicate a separate subgroup or category.

A random-effects meta-analysis of seven studies comprising 284 patients was conducted (Figure 2). The pooled mean serum PCT level among individuals with culture-confirmed melioidosis was 14.46 ng/mL (95% CI: 4.59–24.33). Substantial heterogeneity was observed (*I*^2^ = 87.7%, *p* < 0.0001), likely attributable to differences in assay platforms, study periods, and variability in clinical severity across cohorts. The pooled estimate should be interpreted as a descriptive summary of reported PCT levels rather than a definitive clinical threshold, given substantial inter-study variability.

#### 3.5.2. Sensitivity Analysis

**Figure 3:**
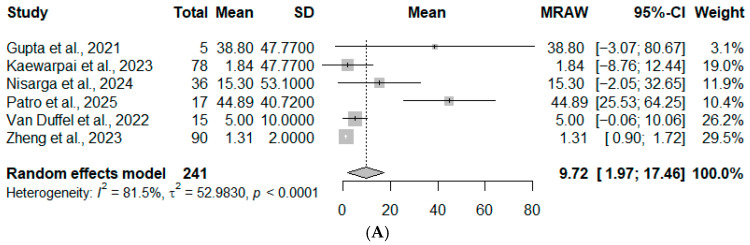

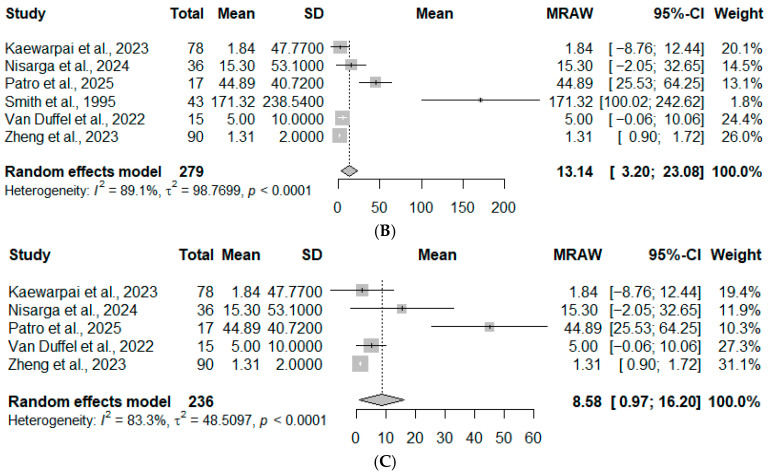
Sensitivity analyses. (**A**) Sensitivity analysis [1,3,8,11,12,13] excluding the historical study by Smith et al. (1995) [6]. (**B**) Sensitivity analysis [1,3,6,8,11,12] excluding the smallest sample study by Gupta et al. (2021) [13]. (**C**) Sensitivity analysis [1,3,8,11,12] excluding both Smith et al. (1995) [6] and Gupta et al. (2021) [13]. Squares represent individual study effect sizes, with horizontal lines indicating 95% confidence intervals. The size of each square reflects the study weight. The diamond represents the pooled estimate from the random-effects model. Bold text is used to highlight the overall summary estimate and does not indicate a separate subgroup or category.

Given the high degree of heterogeneity in the primary meta-analysis, a series of sensitivity analyses was performed to explore the influence of individual studies on the pooled estimate. First, exclusion of the earliest historical study, Smith et al., 1995 [6], reduced the sample to 241 patients and produced a pooled mean PCT level of 9.72 ng/mL (95% CI: 1.91–17.46), with heterogeneity remaining high (*I*^2^ = 81.5%, *p* < 0.0001) (Figure 3A). Next, removal of the smallest study, Gupta et al., 2021 [13], resulted in a pooled mean PCT level of 13.14 ng/mL (95% CI: 3.20–23.08); however, heterogeneity increased (*I*^2^ = 89.1%, *p* < 0.0001) (Figure 3B). Finally, exclusion of both the historical study and the smallest sample, Smith et al., 1995 [6], and Gupta et al., 2021 [13], yielded a pooled mean of 8.58 ng/mL with persistent heterogeneity (*I*^2^ = 83.3%, *p* < 0.0001) (Figure 3C).

Across all analyses, the consistently elevated PCT levels demonstrate that contemporary cohorts offer more homogeneous estimates, suggesting relative consistency of elevated PCT levels across cohorts despite persistent heterogeneity.

### 3.6. Publication Bias

Publication bias was explored using visual inspection of the funnel plot (Supplementary Figure S1) and Egger’s regression test. Although Egger’s test was not statistically significant (*p* = 0.212), formal assessment of small-study effects is unreliable when fewer than 10 studies are included. Therefore, publication bias cannot be definitively excluded, and findings should be interpreted cautiously [14].

## 4. Discussion

This study provides the first pooled quantitative estimate of serum procalcitonin levels in culture-confirmed melioidosis. The findings demonstrate that PCT concentrations are markedly elevated and fall within ranges commonly reported in severe bacterial sepsis and septic shock. This extends current knowledge by providing a reference point for interpreting PCT values in melioidosis, particularly in endemic settings where diagnostic uncertainty and delayed recognition are common. This provides a clinically interpretable reference range that may assist clinicians in distinguishing expected biomarker elevations from atypical values in severe infection.

These findings highlight the biological plausibility of PCT as a biomarker of severe systemic inflammation in *Burkholderia pseudomallei* infection. The magnitude of PCT elevation observed in this review underscores melioidosis as a hyperinflammatory bacterial sepsis, where PCT reflects both bacterial burden and host response. In multiple cohorts, including those from China, India, and Thailand, the highest PCT levels were observed in patients with septic shock or early death, suggesting that PCT reflects the severity of disease rather than being pathogen-specific [1,3,6]. Variations in PCT levels between studies likely stem from host and methodological factors, including differences in age, comorbid diabetes, renal impairment, timing of sampling, infection site, and assay technology. These observations reinforce that PCT should be interpreted within its clinical context and used to complement, rather than replace, established clinical assessments and severity scoring systems.

In healthy individuals, circulating PCT concentrations are typically below 0.05 ng/mL. Most clinical assays report reference ranges between 0.05 and 0.5 ng/mL, depending on analytical sensitivity, with upper detection limits approaching 1000 ng/mL. These methodological variations should be considered when interpreting absolute PCT values across studies [15,16,17,18]. When compared with the broader sepsis literature, the magnitude of PCT elevation observed in melioidosis is broadly comparable to that reported in severe Gram-negative sepsis, where levels frequently exceed 10 ng/mL and may reach > 100 ng/mL in septic shock. These findings suggest that elevated PCT in melioidosis reflects the severity of systemic infection rather than a pathogen-specific response. However, the available data do not allow a definitive conclusion that PCT levels in melioidosis exceed those observed in other forms of Gram-negative sepsis. The early work of Smith et al. (1995) first documented extreme PCT elevations in fatal melioidosis cases [6], a result echoed by more recent studies from India and China showing similarly increased levels among non-survivors and bacteremic patients [1,3]. A contemporary study from Hainan also found that higher PCT correlated with sepsis severity and rainfall-driven infection surges, linking biomarker response with epidemiologic factors [19]. Renal dysfunction, which is common in severe melioidosis, may contribute to elevated PCT levels through reduced clearance and systemic inflammation [20]. As renal function was not consistently reported or adjusted for in the included studies, this represents an important potential confounder. Comparative investigations have shown that *B. pseudomallei* infections produce higher PCT levels than *B. cepacia* or other *Burkholderia* species, suggesting a distinct host–pathogen interaction pattern [8]. Nevertheless, unlike general sepsis, where large multicenter trials have established PCT-guided algorithms for antibiotic management, melioidosis data remain sparse, fragmented, and methodologically heterogeneous. This review, therefore, extends the sepsis biomarker literature into a neglected tropical context, confirming PCT’s utility as a marker of severe systemic illness while underscoring the absence of evidence supporting melioidosis-specific diagnostic or therapeutic thresholds. Importantly, the available evidence does not support the use of PCT as a diagnostic biomarker capable of distinguishing melioidosis from other causes of bacterial sepsis. Instead, PCT appears to reflect the host inflammatory response and overall disease severity. The pooled mean PCT level provides a useful benchmark for clinicians, indicating that markedly elevated PCT values are common in melioidosis and should be interpreted as evidence of severe systemic infection. However, this estimate should not be used as a diagnostic cutoff or prognostic threshold for individual patients. In practice, this may help clinicians recognize that very high PCT levels in melioidosis are expected and should not be misinterpreted as atypical or indicative of alternative diagnoses. The use of pooled raw means rather than log-transformed values may have contributed to residual heterogeneity and limits direct interpretation of central tendency in skewed distributions.

The present study has several methodological strengths that enhance its reliability. It followed a registered protocol, implemented a comprehensive multi-database search, and restricted inclusion to culture-confirmed melioidosis to ensure diagnostic accuracy. The application of random-effects modeling with prespecified sensitivity analyses improved the robustness of pooled estimates by addressing heterogeneity from older assay technologies and small-sample studies. Furthermore, the critical appraisal of study quality using standardized risk-of-bias tools enhances confidence in the synthesized evidence. Nevertheless, important limitations must be acknowledged. The number of eligible studies was limited, reducing the precision and generalizability of pooled estimates. The pooled mean does not capture substantial inter-individual variability in PCT levels, which may limit its application in individual patient-level prognostic assessment and obscure potentially important distributional differences. As individual-level distributions were not consistently reported, it was not possible to estimate the proportion of patients with PCT values exceeding clinically relevant thresholds (e.g., >0.5 ng/mL or >2 ng/mL). The magnitude of pooled PCT values relative to standard reference ranges suggests that the majority of patients likely had elevated concentrations, although precise estimation was not feasible. Given that procalcitonin is likely log-normally distributed, the use of raw means rather than geometric means may have contributed to inflated heterogeneity estimates (*I*^2^) and limits comparability with studies using log-transformed analyses. Significant variability in assay type, timing of sample collection, and definitions of severe disease likely contributed to residual heterogeneity. In this context, geometric means and log-transformed analyses may provide a more appropriate representation of central tendency and dispersion for skewed biomarker data. Although such approaches were considered, consistent application was not feasible due to heterogeneous reporting formats across studies. Future meta-analyses using standardized reporting of log-transformed data may yield more robust and clinically interpretable estimates. Furthermore, consideration of the distribution of PCT values suggests that higher values within the right tail are likely to reflect more severe systemic illness. While formal estimation of this distribution was not possible, the observed magnitude of PCT elevation relative to normal reference ranges indicates that a substantial proportion of patients likely had markedly elevated levels. This variability may itself carry prognostic significance and warrants further investigation in studies with individual-level data.

Most studies reported unadjusted comparisons between survivors and non-survivors and did not control for potential confounders such as baseline organ dysfunction, comorbidities, or treatment variations. Additionally, PCT measurement was often performed selectively in more severely ill patients, introducing possible spectrum bias. The publication bias cannot be ruled out, given the small number of available studies and the predominance of single-center reports. The substantial heterogeneity observed in this meta-analysis likely reflects genuine clinical and methodological differences across studies, including variation in assay platforms, timing of sample collection, disease severity, and patient comorbidities. Therefore, the pooled estimate should be interpreted as a broad summary rather than a precise value applicable across all clinical settings. A quantitative meta-analysis of prognostic associations was not feasible due to heterogeneity in outcome definitions and inconsistent reporting of effect sizes across studies. Most studies reported unadjusted comparisons without sufficient data for pooled analysis. Moreover, in several studies, the assay method used for PCT measurement was not reported. We did not contact study authors to obtain missing information, which limited our ability to fully evaluate assay-related heterogeneity.

Despite these constraints, the results have clear implications for both clinical practice and future research. Clinicians working in endemic regions should recognize that very high PCT levels in suspected melioidosis cases are common and should be interpreted as evidence of severe systemic infection warranting urgent and aggressive management, rather than as a disease-specific diagnostic marker. PCT may serve as a useful adjunct for early risk stratification when interpreted alongside clinical parameters, lactate, and complementary biomarkers such as C-reactive protein (CRP) and interleukin-6 (IL-6) [5,7]. At the research level, these findings highlight an urgent need for large, multicenter, prospective studies using standardized PCT assays, predefined sampling schedules, and harmonized outcome definitions. Future studies should employ multivariable prognostic modeling to determine whether PCT independently predicts mortality and explore whether combining PCT with other inflammatory and metabolic markers enhances predictive accuracy. Addressing these gaps will be critical for validating PCT as an informative biomarker within melioidosis-specific prognostic frameworks and improving clinical risk assessment in endemic settings.

## 5. Conclusions

This systematic review and meta-analysis confirm that serum procalcitonin (PCT) is markedly elevated in patients with culture-confirmed melioidosis and is associated with disease severity, including septic shock and mortality, based on narrative evidence. While PCT is elevated and associated with disease severity, it should be interpreted as a marker of systemic inflammation rather than a disease-specific biomarker. The pooled estimate provides a reference for expected PCT levels in melioidosis but does not establish a clinically actionable threshold. These findings are based on narrative synthesis and do not establish a quantitative prognostic relationship.

These findings should be interpreted in the context of substantial inter-individual variability and methodological heterogeneity across studies. Significant heterogeneity and limited sample sizes further restrict definitive prognostic interpretation. Future large-scale, multicenter studies using standardized assays and multivariable analyses are essential to establish validated thresholds and to define PCT’s independent prognostic role in guiding clinical management and risk stratification in melioidosis.

## Figures and Tables

**Table 1:** Characteristics of the seven included studies.

Author, Year [Ref.]	Country	Study Design	Melioidosis (*n*)	Measurement Assay	Key Findings/PCT Levels
Zheng et al., 2023 [1]	China	Retrospective cohort	90	NR	High mortality associated with septic shock; PCT levels correlated with severity.
Nisarg et al., 2024 [3]	India	Retrospective cohort	36	NR	Investigated predictors of 28-day mortality; PCT included in clinical feature analysis.
Smith et al., 1995 [6]	Thailand	Prospective observational	43	Immunoluminometric assay	Median PCT levels were higher in patients with more severe disease. All groups were higher than the control. Extremely Severe: 1000 ng/mL (IQR 200–800) Severe: 50 ng/ML (IQR 20–80) Mild: 1 ng/mL (IQR 0.5–3) Control: 0.1 ng/mL (IQR 0.01–0.5)
Patro et al., 2025 [8]	India	Retrospective observational	17	NR	Compared *B. cepacia* and *B. pseudomallei*; elevated PCT was identified in *B. pseudomallei* cases.
Kaewarpai et al., 2023 [11]	Thailand	Prospective observational	78	ELISA	Developed biomarker models; PCT was evaluated alongside IL-1R2 for mortality prediction.
Van Duffel et al., 2022 [12]	Multi-center: Cambodia, Congo, Nepal, Sudan	Prospective observational	15	TRACE	Evaluated PCT accuracy for bacterial infections; Melioidosis was identified as a specific cause in the cohort.
Gupta et al., 2021 [13]	India	Retrospective cohort	5	NR	Study focused on osteoarticular melioidosis; PCT used as an inflammatory marker.

ELISA, Enzyme-linked immunosorbent assay; Ref., Reference; IL, interleukin; NR, not reported (assay details were not available in the original publication); PCT, procalcitonin; TRACE, Time-Resolved Amplified Cryptate Emission.

## Data Availability

Extracted data used for the meta-analysis are provided in Supplementary Table S1.

## Supplementary Materials

The following supporting information can be downloaded at: https://www.mdpi.com/article/10.3390/diseases14040119/s1, Supplementary File S1. Search strategies for each database; Supplementary Table S1. Extracted data; Supplementary Table S2. Newcastle–Ottawa scale of included studies; Supplementary Figure S1. Funnel plot.

## Abbreviations

The following abbreviations are used in this manuscript:

CI	Confidence interval
CRP	C-reactive protein
ELISA	Enzyme-linked immunosorbent assay
IL-1R2	Interleukin-1 receptor type 2
MeSH	Medical subheading
PCT	Procalcitonin
PRISMA	Preferred Reporting Items for Systematic Reviews and Meta-Analyses
TRACE	Time-Resolved Amplified Cryptate Emission
