# Supplementary material for: Serum Procalcitonin in Culture-Confirmed Melioidosis: A Systematic Review and Meta-Analysis with Narrative Evaluation of Clinical and Prognostic Implications
Source: Diseases. 2026 Mar 26;14(4):119. doi: 10.3390/diseases14040119 (PMC13114472; doi:10.3390/diseases14040119)
Supplement: Supplementary file 1 [file diseases-14-00119-s001.zip › Supplementary Figure S1.pdf]

Serum Procalcitonin in Culture-Confirmed Melioidosis: A Systematic Review and Meta-Analysis with Narrative Evaluation of Clinical and Prognostic Implications

Jongkonnee Thanasai [1], Chaimongkhon Chanthot [2], Anchalee Chittamma [3], Supphachoke Khemla [4], Atthaphong Phongphithakchai [5], Moragot Chatatikun [6,7], Jitbanjong Tangpong [6,7], Sa-ngob Laklaeng [6] and Wiyada Kwanhian Klangbud [8,9,*]

1 Faculty of Medicine, Mahasarakham University, Mahasarakham 44000, Thailand; jongkonnee@msu.ac.th
2 Project for the Establishment of the Faculty of Medicine, Nakhon Phanom University, Nakhon Phanom 48000, Thailand; chaimongkhon251269@gmail.com
3 Department of Pathology, Faculty of Medicine Ramathibodi Hospital, Mahidol University,
Bangkok 10400, Thailand; anchalee.chi@mahidol.ac.th
4 Division of Infectious Diseases, Department of Internal Medicine, Nakhon Phanom Hospital,
Nakhon Phanom 48000, Thailand; sup.mednkp@gmail.com
5 Nephrology Unit, Division of Internal Medicine, Faculty of Medicine, Prince of Songkla University, Songkhla 90110, Thailand; atthaphong.p@psu.ac.th
6 School of Allied Health Sciences, Walailak University, Nakhon Si Thammarat 80160, Thailand; moragot.ch@wu.ac.th (M.C.); rjitbanj@wu.ac.th (J.T.); sumoun2528@gmail.com (S.-n.L.)
7 Research Excellence Center for Innovation and Health Products (RECIHP), Walailak University,
Nakhon Si Thammarat 80160, Thailand
8 Medical Technology Program, Faculty of Science, Nakhon Phanom University, Nakhon Phanom 48000, Thailand
9 Faculty of Medicine, Nakhon Phanom University, Nakhon Phanom 48000, Thailand
* Correspondence: wiyadakwanhian@gmail.com

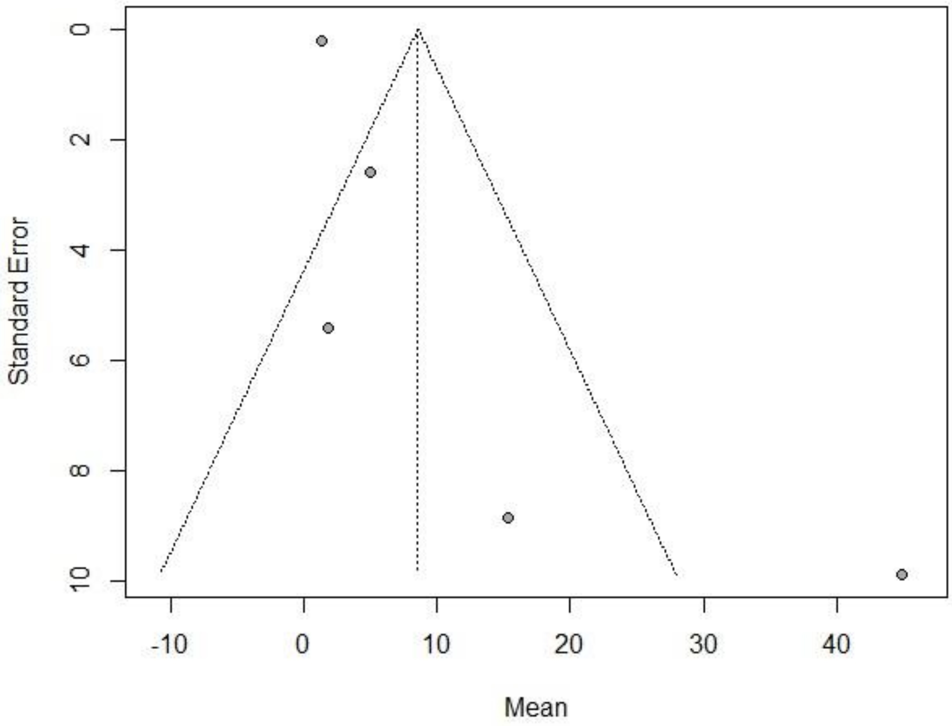

Supplementary Figure S1. Funnel plot
